# Whole transcriptome sequencing analyses of islets reveal ncRNA regulatory networks underlying impaired insulin secretion and increased β-cell mass in high fat diet-induced diabetes mellitus

**DOI:** 10.1371/journal.pone.0300965

**Published:** 2024-04-01

**Authors:** Jinfang Ma, Rui Gao, Qingxing Xie, Xiaohui Pan, Nanwei Tong

**Affiliations:** 1 Department of Endocrinology and Metabolism, West China Hospital, Sichuan University, Chengdu, China; 2 Center for Diabetes and Metabolism Research, West China Hospital, Sichuan University, Chengdu, China; 3 Oxford Centre for Diabetes, Endocrinology and Metabolism, Radcliffe Department of Medicine, University of Oxford, Oxford, United Kingdom; Max Delbruck Centrum fur Molekulare Medizin Berlin Buch, GERMANY

## Abstract

**Aim:**

Our study aims to identify novel non-coding RNA-mRNA regulatory networks associated with β-cell dysfunction and compensatory responses in obesity-related diabetes.

**Methods:**

Glucose metabolism, islet architecture and secretion, and insulin sensitivity were characterized in C57BL/6J mice fed on a 60% high-fat diet (HFD) or control for 24 weeks. Islets were isolated for whole transcriptome sequencing to identify differentially expressed (DE) mRNAs, miRNAs, IncRNAs, and circRNAs. Regulatory networks involving miRNA–mRNA, lncRNA–mRNA, and lncRNA–miRNA–mRNA were constructed and functions were assessed through Gene Ontology (GO) and Kyoto Encyclopedia of Genes and Genomes (KEGG) enrichment analyses.

**Results:**

Despite compensatory hyperinsulinemia and a significant increase in β-cell mass with a slow rate of proliferation, HFD mice exhibited impaired glucose tolerance. In isolated islets, insulin secretion in response to glucose and palmitic acid deteriorated after 24 weeks of HFD. Whole transcriptomic sequencing identified a total of 1324 DE mRNAs, 14 DE miRNAs, 179 DE lncRNAs, and 680 DE circRNAs. Our transcriptomic dataset unveiled several core regulatory axes involved in the impaired insulin secretion in HFD mice, such as miR-6948-5p/*Cacna1c*, miR-6964-3p/*Cacna1b*, miR-3572-5p/*Hk2*, miR-3572-5p/*Cckar* and miR-677-5p/*Camk2d*. Additionally, proliferative and apoptotic targets, including miR-216a-3p/*FKBP5*, miR-670-3p/*Foxo3*, miR-677-5p/*RIPK1*, miR-802-3p/*Smad2* and ENSMUST00000176781/*Caspase9* possibly contribute to the increased β-cell mass in HFD islets. Furthermore, competing endogenous RNAs (ceRNA) regulatory network involving 7 DE miRNAs, 15 DE lncRNAs and 38 DE mRNAs might also participate in the development of HFD-induced diabetes.

**Conclusions:**

The comprehensive whole transcriptomic sequencing revealed novel non-coding RNA-mRNA regulatory networks associated with impaired insulin secretion and increased β-cell mass in obesity-related diabetes.

## Introduction

Type 2 diabetes mellitus (T2DM) primarily arises from a combination of insulin resistance and basal hyperinsulinemia [1], and its global prevalence is on the rise [2].

This metabolic disorder results from the multifaceted interplay among genetic elements, environmental influences, and the combined effects these have on the epigenome [3, 4]. While genetic susceptibility holds considerable sway, substantial attention has also focused on environmental contributors, such as excess nutrition in recent years. Although the cause-and-effect relationship between insulin resistance and hyperinsulinemia remains a subject of debate [5], numerous findings indicated overnutrition promoted insulin resistance and hyperinsulinemia [6–9]. It serves as a precursor to T2DM, followed by a progressive decline in β-cell function and a decrease in the mass of functional β-cell [10]—two critical factors contributing to the development of T2DM. To replicate the human condition of obesity-induced diabetes, high-fat diet, in particular, have been extensively studied for their potential contribution to the development and progression of T2DM [11, 12].

Non-coding RNAs (ncRNAs) are RNA molecules that do not encode proteins, but they interact with protein-coding genes and plays significant roles in various key biological processes [13–15]. Increasing evidence indicated that ncRNAs, including microRNAs (miRNAs), long non-coding RNAs (lncRNAs) and circular RNAs (circRNAs), are crucial regulators in multiple facets of T2DM pathogenesis, such as insulin synthesis, glucose metabolism and homeostasis [15–20]. For example, the downregulation of miR146a enhances *NFkB* -mediated inflammatory events and induces β-cell apoptosis [21], and altered miR-124a expression also contributes to β-cell dysfunction in T2DM [22]. In addition, diminished expression levels of lncRNAs TTC28-AS1 and SNHG17 have been reported to be associated with T2DM susceptibility, showing significant correlation to metabolic features [23], while overexpression of lncRNA-p3134 maintains β-cell mass by providing a protective effect against glucotoxicity-mediated apoptosis [24]. Besides, hsa_circ_0054633 and hsa_circ_0071106 have also been found as diagnostics biomarkers of pre-diabetes and T2DM [25–27]. Recent research findings propose that lncRNAs and circRNAs may serve as miRNA "sponges" or "decoys," engaging in competitive binding with miRNAs, thereby diminishing the regulatory influence of these miRNAs on their intended mRNA targets [15, 28, 29]. Due to the competitiveness, these lncRNAs and circRNAs are also called competing endogenous RNAs (ceRNAs). While a growing body of recent research has centred on the regulation of ceRNAs interactions, especially in cancer, the number of functionally well-annotated ncRNAs related to T2DM remains limited. Moreover, the intricate network of how ncRNAs and mRNAs mutually regulate each other in a model of obesity-related diabetes is yet to be fully elucidated. However, the existing studies mentioned above were derived from analyzing peripheral blood samples and β-cell lines, or were solely profiled in a microarray focusing on a single type of ncRNA. Furthermore, acute toxic injuries in β-cells, such as fatty acid-induced dysfunction, might not serve as an ideal research model for understanding the natural progression of T2DM in humans.

In recent years, whole transcriptome analysis through total RNA sequencing has emerged as an efficient tool for providing a comprehensive view of the transcriptome’s complexity. In our present study, we utilised whole transcriptome sequencing data to establish ncRNA networks within islet tissue, encompassing miRNA–mRNA, lncRNA–mRNA and lncRNA–miRNA–mRNA interactions, using a diet-induced obesity and diabetes mellitus model. Our study aims to correlate transcriptomic features with phenotypes, and systematically unveil pathogenic pathways underlying islet dysfunction and diabetes progression, thereby identifying novel therapeutic targets associated with T2DM.

## Materials and methods

### Mice

Five-week-old male C57BL/6J mice were purchased from GemPharmatech Co., Ltd. (Jiangsu). The mice were given *ad libitum* access to water and food, and were housed in a 12 h light/ dark cycle at an ambient temperature of 22±2°C. Following 1 weeks of acclimatization, the mice were fed either a high-fat diet (HFD) comprising 60% fat, 20% protein and 20% carbohydrate (D12492, Jiangsu Synergetic Bioengineering Co., Ltd.) or a chow diet (CD) that contained 10% fat, 20% protein and 70% carbohydrate (D12450 J, Jiangsu Synergetic Bioengineering Co., Ltd.). After 24 weeks of dietary intervention, intraperitoneal glucose tolerance tests (IPGTT), intraperitoneal insulin tolerance tests (IPITT) and glucose stimulated insulin secretion test (GSIS) were performed in both HFD and CD mice. Following a one-week recovery period, the body weights of all mice were measured, and serum samples were collected. Islets were then isolated from both HFD and CD mice for further sequencing at the end of the dietary intervention.

Daily health checks were conducted throughout the study to promptly address any signs of distress. Experimental protocols adhered to the "3Rs" principle, minimizing animal usage and maximizing welfare. Cervical dislocation was employed for humane euthanasia of mice. This rapid method was executed by trained personnel to ensure immediate loss of consciousness and death. All procedures were carried out in accordance with the National Institutes of Health Guidelines for the Care and Use of Animals (IACUC) and had been approved by the Institutional Animal Care and Use Committee at Sichuan University.

### In vivo dynamic physiological tests

IPGTT: Mice underwent a 16 h fasting period before receiving an intraperitoneal (i.p.) injection of D-glucose (2 g/kg). Tail blood glucose levels were measured at 0, 15, 30, 60, 90, and 120 min after the injection using a glucometer (Roche, Basel, Switzerland).

#### GSIS

Mice underwent a 16 h fasting period prior to an i.p. injection of D-glucose (2 g/kg). At 0, 15, 30, and 120 min post-injection, 25 μL of blood was collected from the tail vein to measure insulin levels subsequently.

#### IPITT

Mice were fasted for 6h before receiving an i.p. injection of insulin (0.75 U/kg). Tail blood glucose levels were measured at 0, 15, 30, 45, 60, and 90 min post-insulin injection with a glucometer.

### In vivo biochemical measurements

Serum samples were obtained by centrifuging the blood samples at 5000 rpm for 30 min and stored at -80°C. Serum insulin levels were measured using an Ultra-sensitive Mouse Insulin ELISA Kit (Crystal Chem, Chicago, IL). Serum total cholesterol (TC), triglyceride (TG), aspartate aminotransferase (AST), alanine aminotransferase (ALT), and low-density lipoprotein-cholesterol (LDL-C) were determined after a 16 h fasting period using an automatic biochemical analyzer (HITACHI 7100, Hitachi Koki Co. Ltd., Japan).

### Immunohistochemistry of pancreatic tissues

Immunohistochemical staining was performed to examine pancreatic islet cells according to standard protocols. In brief, pancreatic tissues were fixed overnight with 4% paraformaldehyde (PFA). The fixed tissue samples were embedded in paraffin, sliced into sections, and subsequently subjected to baking, dewaxing, and hydration. Then, slides were heated in IHCTek epitope retrieval solution for 30 min and permeabilized with 0.25% Triton X-100 in PBS for 10 min. The samples were then blocked with 5% goat serum in PBS and incubated overnight at 4°C with anti-insulin (1:500 dilution, Servicebio, Wuhan, China) or anti-glucagon antibody (1:500 dilution, Servicebio) or anti-Ki67 (1:1000 dilution, Servicebio). After thorough PBS washes, the slides were incubated for 1 h at room temperature with a mixture of Alexa Fluor 555- or Alexa Fluor 488-conjugated secondary antibodies (1:500 dilution, Servicebio). All sections were counterstained and mounted using ProLong Gold antifade reagent with DAPI (Servicebio). Negative controls were obtained by omitting the primary antibodies. Images were then captured using a Nikon microscope (Eclipse C1).

### TUNEL staining of pancreatic tissues

The sliced pancreatic tissues were first dried in a 60°C oven for 30 min and then dewaxed with xylene (5 min × 3 times) followed by dehydration using a series of ethanol washes (100% ethanol, 95% ethanol, and 70% ethanol; each repeated three times). The sections were then incubated with protein kinase K for another 30 min. After rinsing with PBS, the terminal deoxyribonucleotide transferase TdT and luciferase-labeled dUTP were added, and the reaction was incubated at 37°C for 1 h. The sections were subsequently incubated with HRP-specific antibodies for 1 h at 37°C in an incubator. Finally, 3,3’-Diaminobenzidine (DAB) was added as the substrate, and the reaction was allowed to proceed at room temperature for 10 min. Following the staining of nuclei with hematoxylin, images were captured using a Nikon microscope (Eclipse C1), and the number of cells was manually counted.

### Hematoxylin-eosin (HE) staining of liver tissues

The sliced liver tissues were prepared as previously described [30], and they were subsequently immersed in a 5% hematoxylin aqueous solution and stained for 5 min. After rinsing with running water, the stained samples were incubated in a hematoxylin differentiation solution for 15 s, followed by treatment with hematoxylin scott tap bluing for 15 s. The sections were rinsed again with running water, immersed in eosin (0.5%) staining for 3 min, and subjected to a final rinse with running water. The samples were then dehydrated, cleared, mounted, and examined.

### Oil red O staining

For assessing lipid droplet formation, frozen sections of liver tissue were stained with Oil Red O for 30 minutes and subsequently counter-stained with haematoxylin for 1 minute. The stained frozen sections were then washed, dehydrated, and mounted for imaging.

### Islet isolation

Islets were isolated from both HFD and CD mice with pancreas extraction following intra-ductal injection with collagenase P (Roche, Basel, Switzerland) in Hank’s Balanced Salt Solution (Sigma). After three rounds of hand selection under a light microscope, islets were collected for further experiments.

### In vitro insulin secretion measurements

Insulin secretion was performed by static incubation as described earlier [12]. Briefly, islets were isolated from 4–5 mice per group and cultured overnight. On the following day, groups consisting of 20 size-matched islets were pre-incubated in a custom-made KRB buffer (pH 7.4) for 1 hour. Subsequently, these islets were exposed to either 2 or 20 mmol/L glucose, optionally combined with 0.5 mM palmitate acid (PA), for an additional hour at 37°C under 5% CO2. After this treatment, supernatants were harvested, and islets were lysed in an acid ethanol solution. Insulin within both supernatants and contents were measured by ELISA kits (Mercodia, Sweden). The secreted insulin was calculated as percentage of total contents per hour.

### RNA sequencing

Total RNA was isolated from handpicked islets of both HFD and CD mice (each group consisted of three biological replicates, with each replicate derived from the islets of 3 mice) using TRIzol (Invitrogen, Carlsbad, CA, USA). RNA degradation and contamination were monitored on 1% agarose gels. Subsequently, the quality of the isolated RNA samples was evaluated using an Agilent Bioanalyzer 2100 (Agilent Technologies, Santa Clara, CA, USA), and the quantity was measured using a NanoDrop ND-2000 (NanoDrop Technologies, USA). Only RNA sample meeting high-quality standards (OD260/280 = 1.8~2.2, OD260/230≥2.0, RIN≥8.0, 28S:18S≥1.0, >5μg) were utilized to construct sequencing libraries.

RNA purification, reverse transcription, library construction, and sequencing were all carried out at Shanghai Majorbio Bio-pharm Biotechnology Co., Ltd. (Shanghai, China), adhering to the manufacturer’s instructions from Illumina (San Diego, CA). For RNA-seq transcriptome strand library preparation, 1 μg of total RNA was utilized following the TruSeqTM Stranded Total RNA Library Prep Kit protocol by Illumina. Shortly, ribosomal RNA (rRNA) depletion instead of poly(A) purification was performed using the Ribo-off rRNA Depletion kit. Subsequently, the RNA underwent fragmentation using a fragmentation buffer. Thereafter, first-strand cDNA synthesis took place with random hexamer primers. The RNA template was then removed, and a replacement strand was synthesized, incorporating dUTP in lieu of dTTP to generate ds cDNA. The presence of dUTP effectively quenched the second strand during amplification since polymerase bypassing this nucleotide was inhibited. AMPure XP beads were employed to separate ds cDNA from the second strand reaction mixture. To prevent self-ligation during adapter ligation, a single ’A’ nucleotide was added to the 3’ ends of these blunt fragments. Finally, multiple indexing adapters were ligated to the ends of the ds cDNA. Libraries were size-selected for cDNA target fragments ranging between 200–300 bp on a 2% Low Range Ultra Agarose, followed by PCR amplification for 15 cycles using Phusion DNA polymerase (NEB). Upon quantification with TBS380, the paired-end RNA-seq sequencing library was sequenced on an Illumina NovaSeq6000 sequencer (2 × 150bp read length). In parallel, for small RNA library preparation, a total of 3 μg of total RNA per sample was used as input material. Sequencing libraries were constructed using the TruSeq TM Small RNA Sample Prep Kit from Illumina according to the manufacturer’s guidelines. Activated 5’ and 3’ adaptors were ligated to the total RNA, respectively. The adaptor-ligated RNA was subsequently converted into first-strand cDNA via reverse transcription utilizing reverse transcriptase and random primer. A PCR reaction was conducted with primers complementary to the two adaptors for 11–12 cycles. Fragments of the appropriate size were isolated through a 6% Novex TBE PAGE gel. Once quantified with TBS380, the single-end RNA-seq sequencing library was sequenced on an Illumina NovaSeq 6000 sequencer. For the longRNA-seq dataset, each of the six samples produced over 17.04 gigabases (Gb) of high-quality (Clean) data, amounting to a collective total of 107.84 Gb with a consistently high Q30 base percentage exceeding 94.78%. In contrast, for the smallRNA-seq experiment, we obtained a total of 73.65 million (M) raw sequencing reads across the six samples, with every individual sample yielding more than 11.07 M raw reads. These samples maintained a robust Q30 base call rate of at least 94.99%.

The quality of the sequencing data was evaluated with fastx_toolkit (Version 0.0.14) and fastp (Version 0.19.5). Subsequently, reads were aligned to the mouse reference genome (GRCm39) using Bowtie2 (Version 2.2.9) and HISAT2 (Version 2.1.0). The mapped reads from the two libraries were then assembled using StringTie (Version 1.3.3b) and cufflinks (Version 2.2.1). Gene expression levels were quantified by RSEM (Version 1.3.1) and were normalized by the method of transcripts Per Million reads (TPM). Differentially expressed mRNA transcripts (DEMs) between HFD and CD groups were identified based on criteria of log_2_FC > 1 or < -1 and P-value < 0.05, performed by DEseq2 package (Version 1.10.1).

### Identification of miRNAs

Mapped small RNA tags were first employed to identify known miRNAs using the miRBase22.0 database (http://www.mirbase.org/). The modified miRDeep2 software (https://www.mdc-berlin.de/content/mirdeep2-documentation) was utilized to retrieve potential miRNAs and generate their secondary structures. Custom scripts were employed to derive both the miRNA counts and the base bias at the first position of identified miRNAs with specific lengths, as well as at each position across all identified miRNAs, respectively. Subsequently, the small RNA tags were aligned against both the Rfam database and the Repbase database, filtered ribosomal RNA (rRNA), transfer RNA (tRNA), small nuclear RNA (snRNA), small nucleolar RNA (snoRNA) and other ncRNA and repeats. The miRDeep2 software was further used to predict novel miRNAs based on Dicer cleavage sites and the minimum free energy of the unannotated small RNA tags from previous steps. The expression levels of each miRNA were calculated using the transcripts per million reads (TPM) method. Significant DE miRNAs were extracted with |log2FC| >1 and P-value < 0.05.

### Identification of lncRNAs

We initially refer to the known lncRNAs catalogued in NONCODE (http://www.noncode.org/index.php). Subsequently, we discarded transcripts that overlapped with known protein-coding genes on the same strand, transcripts with length shorter than 200 nt, the open reading frame (ORF) longer than 300 nt, and an exon number of less than 2. Next, we employed the Coding Potential Calculator (CPC), Coding-Non-Coding index (CNCI), and Coding Potential Assessment Tool (CPAT) to filter out transcripts demonstrating coding potential (with CPC score < 0.5; CNCI score < 0; CPAT score < 0.5). Furthermore, the remaining transcripts harboring known protein domains were eliminated by Pfam Scan according to Pfam HMM. The surviving transcripts were thus classified as reliably expressed lncRNAs, which were further categorized into intergenic, sense exon overlap, antisense, sense intron overlap, and bidirectional lncRNA types. To identify DE lncRNAs between two distinct groups, the expression levels of each lncRNA were calculated based on the transcripts per million reads (TPM) method. RSEM was utilized for quantifying lncRNA abundances. Fundamentally, differential expression analysis was conducted using DESeq2, LncRNAs with |log2FC| ≥ 1 and P-value < 0.05 were considered significantly differentially expressed.

### Identification of circRNAs

The CIRI (CircRNA Identifier) and find_circ tools were employed to identify circRNAs. Subsequently, the identified circRNAs were categorized into exon, intron, and intergenic circRNAs. For the purpose of identifying DE circRNAs between two different groups, the expression level of each circRNA was calculated based on the reads per million mapped reads (RPM) method. Essentially, differential expression analysis was carried out using DEGseq, where circRNAs with |log2 FC| ≥ 1 and P-value < 0.05 were deemed significantly differentially expressed.

### Quantitative real-time PCR

To validate the reliability of the RNA-sequencing data, quantitative real-time PCR (qRT-PCR) was conducted. RNA was extracted from islets using the AxyPrep Total RNA Mini Kit (Axygen, Corning, New York, USA) according to the manufacturer’s instructions. cDNA was synthesized from 0.5 μg of RNA using oligo(dT) and random hexamer primers with reverse transcriptase (Takara RT Kit). Quantitative PCR was performed using the CFX96 system (Bio-Rad) and SYBR Green Master Mix (Bio-Rad). The primer sequences are provided in Table 1.

**Table 1:** The primers used in this research.

Gene	Primer (Forward)	Primer (Reverse)
Asb11	CGATCCCCACTCCATGAGG	ACAAGATTGACGTTGATGCCTT
Cpa1	GTCTTCGGCAATGAGAACTTTGT	GGAAGGGCACTCGAACATCG
Cpb1	TGATGGCAACCGGGTGTTC	TTGGTGTTGGCTAGTTCCTGA
Ctrb1	ATGGCATTCCTTTGGCTTGTG	GGATAGCATCCTCTCCGTTGAC
Ctrl	AGCCTAACCCTTAGCCTGGTC	TCCCCGTTGACAATTCTCTGA
Erp27	CAGCGGCAAAAGGGAAAATGG	GGCAGTGTGTCCCACTTGTC
β-Actin	CTAAGGCCAACCGTGAAAAG	ACCAGAGGCATACAGGGACA

### GO and COG analysis

Gene Ontology (GO) analysis was employed to investigate the primary function of the differential expressed mRNAs. GO categories, derived from the Gene Ontology (http://www.geneontology.org/). describe attributes of gene products and provide the gene regulatory network based on biological processes, molecular functions and cellular components.

The Clusters of Orthologous Groups (COGs) were constructed by applying the criterion of consistency of genome-specific best hits to the results of an exhaustive comparison of all protein sequences from the genomes of bacteria, archaea and eukaryotes [31]. This COGs database (http://www.ncbi.nlm.nih.gov/COG) was then employed to facilitate applied to functional and phylogenetic annotation of the sequenced genomes in our study.

### KEGG pathway analysis

Pathway analyses of differentially expressed mRNAs were conducted using the latest Kyoto Encyclopedia of Genes and Genomes (KEGG) database (https://www.genome.jp/kegg/), allowing us to identify the specific biological pathways in which the significantly enriched mRNAs were involved.

### Prediction of miRNA target gene

To predict the differentially expressed mRNAs targeted by the differentially expressed miRNAs, the miRanda (http://www.miranda.org/) algorithms were employed to identify miRNA binding sites. The mRNAs associated with the differentially expressed miRNA were annotated using both the GO and the KEGG pathway databases.

### Target gene prediction and functional analysis of lncRNAs

To investigate the functions of the identified lncRNAs, we predicted their cis-target genes (neighboring genes). We accomplished this by selecting coding genes located within a 10,000 bp range both upstream and downstream of the identified lncRNAs using a Python script. Subsequently, we annotated the genes associated with the differentially expressed lncRNAs using the GO and the KEGG pathway databases.

### Overview of the processes used to identify ceRNA interaction pairs

Based on the expression levels of mRNAs, miRNAs, and lncRNAs or circRNAs, Pearson’s correlation coefficient and p value were calculated for miRNA-target (predicted via miRanda). Pairs with negative correlations and *p*-value < 0.05 were selected for further analyses and the co-expression relationships were visualized using Cytoscape (v3.10.0).

### Statistical analysis

GraphPad Prism 8.0 (San Diego, CA, USA) was utilized to perform statistical analyses. Data are expressed as mean ± standard error of the mean (SEM). We used two-tailed unpaired *t*-tests for comparison between two groups, one-way ANOVA and two-way ANOVA with repeated measures for comparison involving three or more groups, followed by Bonferroni’s post-hoc test. A two-tailed p-value < 0.05 was considered statistically significant.

## Results

### General characteristics and impaired glucose homeostasis and islet function in HFD mice

**Fig 1:**
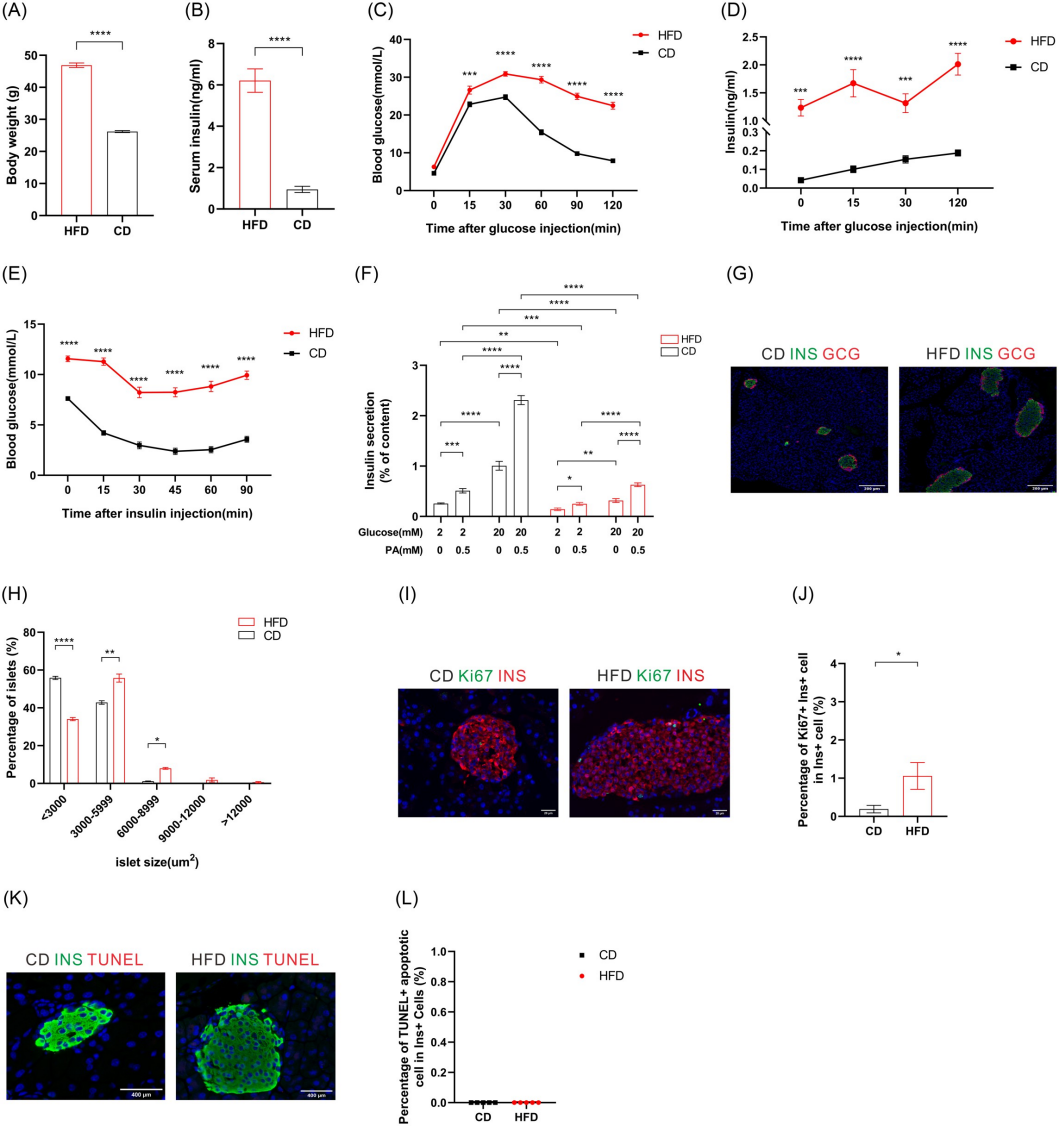
Glucose metabolism disorders and islet dysfunction of HFD mice. (A) The average body weights of high-fat diet (HFD) and chow diet (CD) mice. (n≥ 23 mice /group). (B) The average serum insulin levels of HFD and CD mice. (n≥14 mice /group). (C-D) Blood glucose (C) and the corresponding plasma insulin concentration (D) during IPGTT. (n≥10 mice/group). (E) Blood glucose during IPITT. (N≥20 mice/group). (F) Insulin secretion in islets isolated from HFD and CD mice. (n = 7 samples/group, n = 3–4 mice/group). (G) Representative immunofluorescence staining of pancreatic islets from HFD and CD mice. Red indicates glucagon-positive cells, and green indicates insulin-positive cells. (H) Islet size distributions analyzed by morphometry (N = 4 mice/group). (I) Representative immunofluorescence staining showing Ki67+ Ins+ cells in pancreatic sections. Red indicates insulin-positive cells, and green indicates Ki67-positive cells. (J) Quantification of the percentage of Ki67+ Ins+ cells in total Ins+ cells. (n = 9 islets/group, n = 3 mice/group). (K) Apoptotic signals at the pancreatic islets of HFD and CD mice. Green indicates insulin-positive cells, and red indicates apoptosis signals detected by the TUNEL method. (L) Quantification of the percentage TUNEL+ apoptotic cell in total Ins+ cells. (n = 5 islets/group, n = 3 mice/group). Data presented as mean ± SEM. *p<0.05, **p<0.01, ***p<0.005, **** p<0.001.

The long-term HFD is recognized for inducing a mouse model for obesity-related diabetes. To demonstrate the comprehensive metabolic characteristics in this HFD model, we investigated the profile of general characteristics, glucose metabolism, islet secretion, and architecture in C57BL/6J mice fed either a 60% HFD or a chow diet (CD) over a 24-week period. Severe obesity and hyperinsulinemia were observed in HFD mice, as compared to the CD mice (Fig 1A and 1B). Moreover, S1 Fig demonstrates that the HFD induced fatty liver, hyperlipidemia, and hepatic toxicity. This is manifested by cytoplasmic ballooning and lipid accumulation observed through HE staining and Oil Red O staining (S1A and S1B Fig), along with significantly elevated plasma levels of TC (p < 0.0001), TG (p<0.01), LDL-C (p<0.001), AST (p<0.0001), and ALT (p<0.001) shown in S1C–S1G Fig.

To evaluate islet function, we performed IPGTT, GSIS and IPITT. The results revealed that HFD mice displayed a marked deterioration in glucose tolerance compared to CD mice, as evidenced by significantly elevated peak and a slower decay of plasma glucose level following glucose administration (Fig 1C). Besides, throughout GSIS, HFD mice consistently displayed significantly higher insulin levels compared to CD mice at every measured time point, despite CD mice showing an appropriate insulin secretory response to the glucose challenge (Fig 1D). ITTs were subsequently conducted to evaluate systemic insulin sensitivity, and our finding showed a marked insulin resistance following 24 weeks of HFD treatment. The blood glucose levels in HFD mice were significantly higher at 0, 15, 30, 45, 60, and 90 min post-insulin injection (Fig 1E), compared to CD mice.

We also evaluated insulin secretion in intact islets isolated from HFD mice or CD mice, under conditions of low and high glucose concentrations, and in the presence and absence of PA. The insulin secretion of islets from HFD mice was notably lower than that from CD mice, regardless of whether stimulatory glucose or PA was present, suggesting impairment of β-cell function. When comparing the fold increase in insulin secretion due to 20 mM glucose stimulation alone, we observed a reduction from 3.91-fold in CD mice to 2.2-fold in HFD mice. Similarly, upon 0.5 mM PA treatment alone, the fold increase dropped from 1.98 in CD mice to 1.74 in HFD mice. The potentiating effect of PA on glucose-stimulated insulin secretion was also found to be impaired in HFD mice (2.52-fold in HFD versus 4.53-fold in CD), which consistent with the previous research [32] (Fig 1F).

Immunofluorescent staining and TUNEL assays were performed to examine proliferative and apoptotic changes in the islet from HFD mice. Our observation revealed a notable increase in the prevalence of large-sized islets (6000–8999 um^2^) in HFD group (Fig 1G and 1H, 7.99% in HFD versus 1.18% in CD) and a higher percentage of Ki67+ Ins+ β-cells within HFD islet (Fig 1I and 1J, 1.06% in HFD versus 0.19% in CD). However, as marked by TUNEL assay, no apoptotic signals (red in the nucleus) were observed in β-cells from both HFD and CD mice (Fig 1K and 1L), suggesting that neither diet led to a significant level of apoptosis.

Hence, our data confirmed the occurrence of impaired glucose homeostasis and compromised islet function in the HFD mice, suggesting the successful establishment of a diet-induced obesity and diabetes mouse model.

### Whole transcriptomic profiles and pathway of islets in diet-induced diabetes

**Fig 2:**
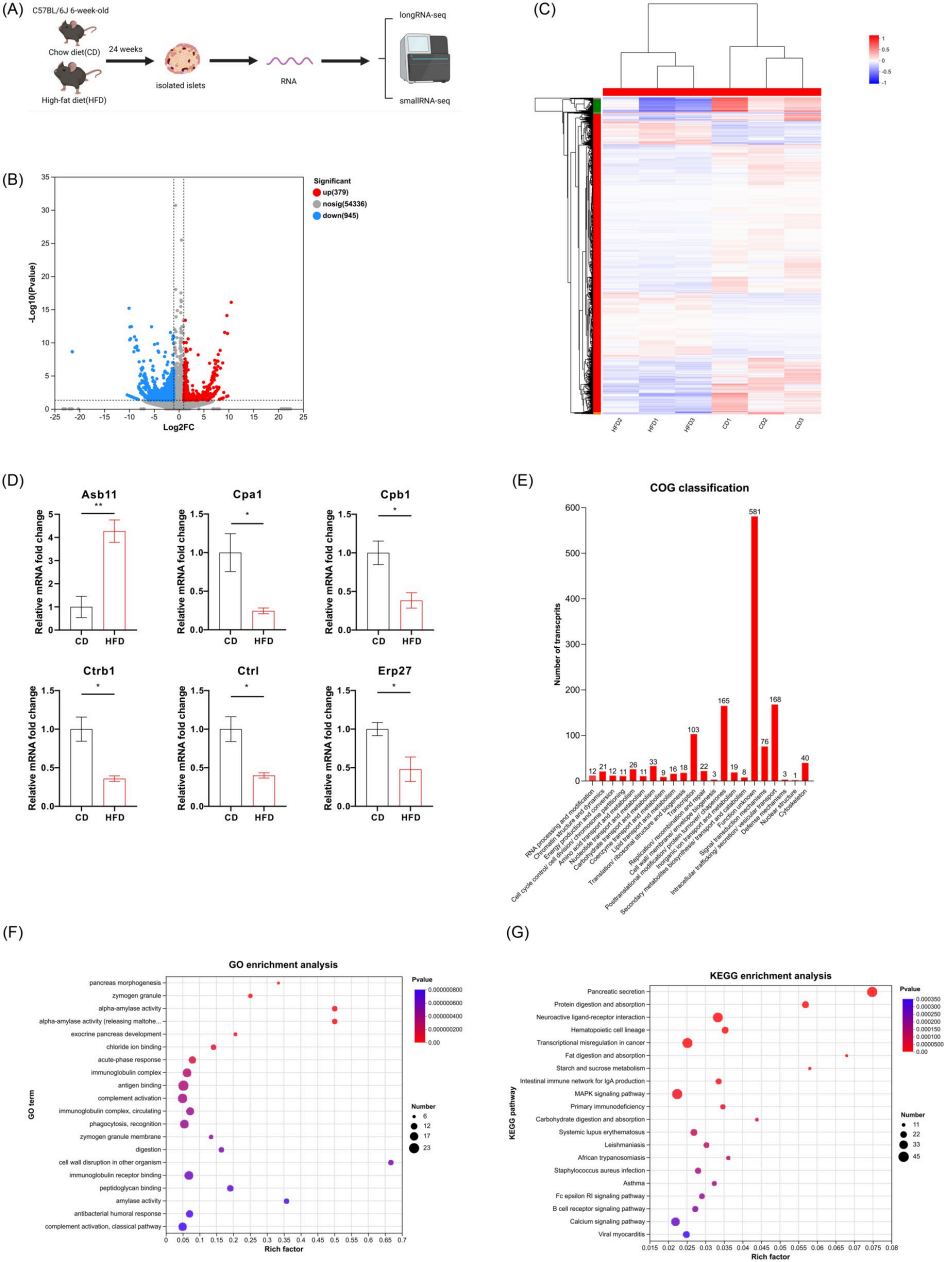
The expressional pattern and function analysis of differentially expressed mRNAs from HFD and CD islets. (A) Schematic of the experimental design for whole transcriptome sequencing. Created with BioRender.com. (B) A volcano plot of the DEMs between the HFD and CD groups. The numbers of upregulated (red dots) and downregulated (blue dots) genes are marked in the graph. (C) Heatmap and hierarchical clustering analyses of the 1324 differentially expressed mRNAs. (D) Random selection of 6 DEMs for the validation of islet transcriptomic data. Data presented as mean ± SEM. *p<0.05, **p<0.01. (E) COG function classification of the DEMs. (F) GO enrichment analysis of DEMs. (G) KEGG enrichment analysis of DEMs. Abbreviations: DEMs, differentially expressed mRNAs; COG, Cluster of Orthologous Groups; GO, gene ontology; KEGG, Kyoto Encyclopedia of Genes and Genomes.

Islets obtained from C57BL/6Jmice fed a 60% HFD or a CD for 24 weeks were isolated for whole transcriptome sequencing (Fig 2A). Following data processing, a total of 1324 differentially expressed mRNAs (DEMs) were identified between the two groups (S1 Table). Among these, 379 showed significant upregulation with a log2 FC≥1, while 945 were notably downregulated with a log2 FC≤-1, as illustrated in the volcano plot (Fig 2B). Heatmap and hierarchical clustering analyses in Fig 2C presented 1324 differentially expressed mRNAs between HFD and CD group. Notably, the expression patterns of DEMs among the three samples within each group showed remarkable similarity, suggesting high reproducibility (Fig 2C). To validate our transcriptomic data, we randomly chose 6 DEMs for assessment via qRT-PCR in a separate set of islet samples derived from both HFD and CD mice. The results showed similar expression patterns to those observed in RNA-sequencing data (Fig 2D).

To elucidate the molecular mechanisms contributing to the changes in these DEMs during diabetes progression, functional analyses were conducted. The Cluster of Orthologous Groups (COG) database was utilized to classify the DEMs, revealing predominant functional clusters related to crucial cellular processes such as intracellular trafficking, vesicular transport mechanisms, and secretion (COG category U). Additionally, there was a notable concentration of DEMs associated with posttranslational modification, protein turnover, and chaperone functions within COG category O (Fig 2E).

The GO enrichment analysis revealed significant enrichment of DEMs in biological processes such as pancreas morphogenesis and exocrine pancreas development, which are crucial pathways related to β-cell differentiation and formation (Fig 2F). We also mapped the DEMs in the KEGG database, resulting in 318 enriched pathways. Notably, the pathways of pancreatic secretion and calcium signaling, which are associated with the islet function and progression of diabetes, were significantly enriched, featuring among the top 20 pathways (Fig 2G).

**Fig 3:**
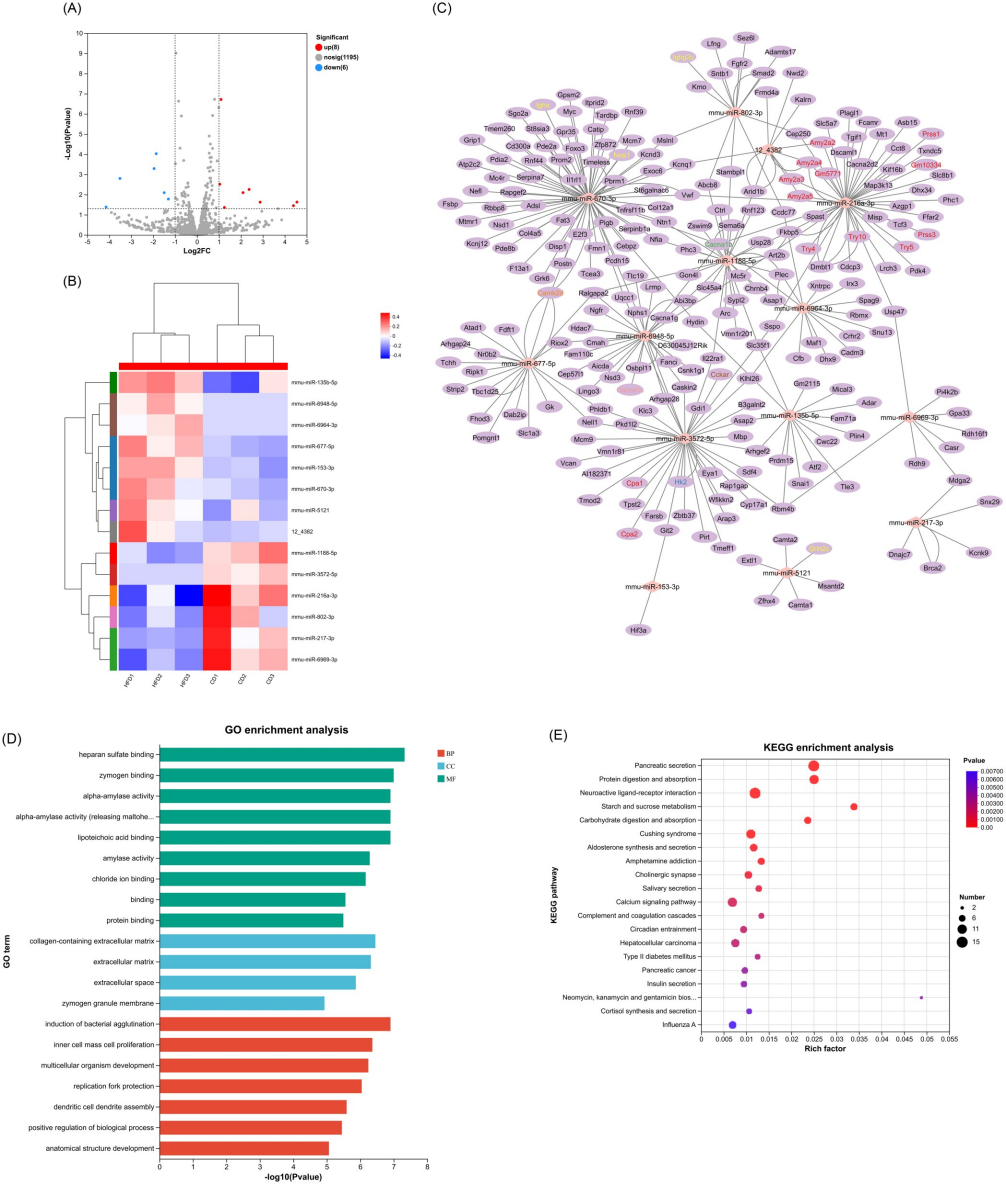
Novel miRNA target identification and function analysis from HFD and CD islets. (A-B) Differentially expressed miRNAs were exhibited by volcanoplot and clustering analysis. (C) miRNAs-mRNAs regulatory network analysis of DE miRNAs and DE mRNAs. The diamond represents DE miRNAs, and ellipses represent DE mRNAs. Target mRNAs associated with key pathways have been color-coded for clarity: red for pancreatic secretion pathway; blue for type 2 diabetes mellitus pathway; yellow for calcium signaling pathway. To denote involvement in multiple pathways, mRNAs are marked green if they participate in both calcium signaling and type 2 diabetes mellitus pathways, orange for those involved in calcium signaling and insulin secretion pathways, pink for mRNAs implicated in all three pathways (calcium signaling, type 2 diabetes mellitus, and insulin secretion), and brown for targets participating in pancreatic secretion, calcium signaling, and insulin secretion pathways. (D) GO enrichment analysis of DE miRNAs-targeted DE mRNAs. (E) KEGG enrichment analysis of DE miRNAs-targeted DE mRNAs.

**Fig 4:**
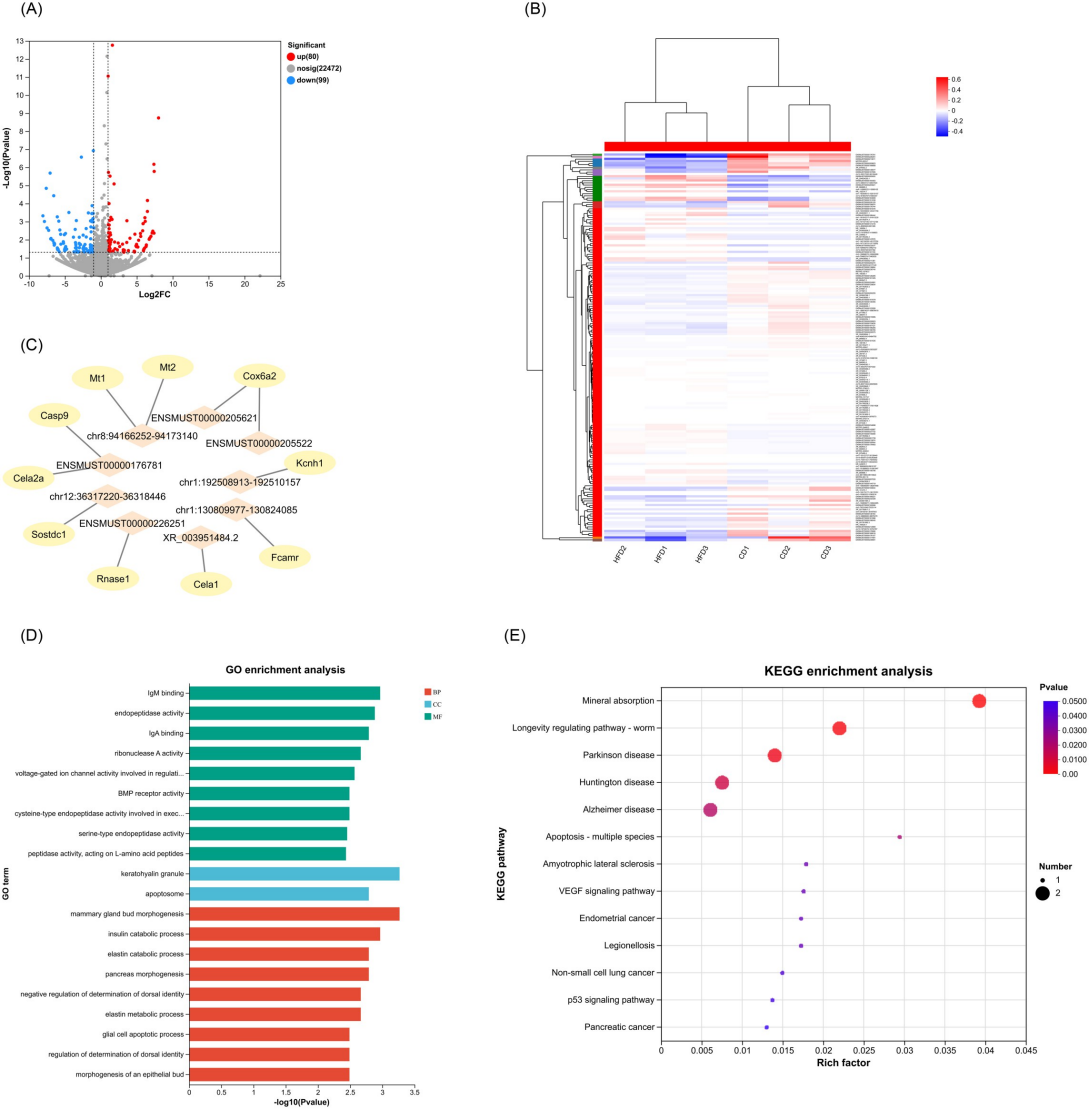
Identification and function analysis of cis target genes of DE lncRNAs in islets. (A-B) Differentially expressed lncRNAs were exhibited by volcanoplot and clustering analysis. (C) LncRNA-cis target genes regulatory network analysis of DE lncRNAs and DE mRNAs. The diamond represents DE lncRNAs, and ellipses represent their corresponding differentially expressed potential cis target genes. (D) GO enrichment analysis of the corresponding differentially expressed potential cis target genes of DE lncRNAs. (E) KEGG enrichment analysis of the corresponding differentially expressed potential cis target genes of DE lncRNAs.

A total of 14 differentially expressed (DE) miRNAs (8 upregulated and 6 downregulated) and 179 DE lncRNAs (80 upregulated and 99 downregulated) were also identified (Figs 3A and 4A), and details regarding these DE miRNAs and DE lncRNAs can be found in S2 and S3 Tables, respectively. The upregulated miRNAs included miR-135b-5p, mmu-miR-153-3p, mmu-miR-5121, mmu-miR-670-3p, mmu-miR-677-5p, mmu-miR-6948-5p, mmu-miR-6964-3p and 12_4382, while the downregulated annotated miRNAs were mmu-miR-1188-5p, mmu-miR-216a-3p, mmu-miR-217-3p, mmu-miR-3572-5p, mmu-miR-6969-3p and mmu-miR-802-3p. These differentially expressed miRNAs were identified through hierarchical clustering analysis shown in Fig 3B. Among differentially expressed (DE) lncRNAs, the most significantly upregulated annotated transcripts included: chr7:140134538–140137224 with the corresponding lncRNA gene ID ENSMUSG00000025464 and gene name Paox; MSTRG.581.10 linked to ENSMUSG00000084799 and gene Ino80dos; chr18:68340371–68342443 associated with ENSMUSG00000007480 and Mc5r; chr4:98115993–98118843 related to ENSMUSG00000028565 and Nfia; and chr7:101899953–101901847 connected to ENSMUSG00000030649 and Anapc15. On the contrary, the most downregulated annotated lncRNAs were chr4:138206284–138207058 from ENSMUSG00000028760 (Eif4g3); ENSMUST00000186785 corresponding to ENSMUSG00000073538 (E330020D12Rik); ENSMUST00000138164 tied to ENSMUSG00000085510 (Mir217hg); chr12:38869930–38870479 linked with ENSMUSG00000004151 (Etv1); and NR_168300.1 which is affiliated with ENSMUSG00000100627 (A830008E24Rik). These differentially expressed lncRNAs were identified via hierarchical clusteringanalysis (Fig 4B).

Moreover, a total of 680 DE circRNAs were discovered, consisting of 357 upregulated circRNAs (347 annotated and 10 non-annotated) and 323 downregulated circRNAs (311 annotated and 12 non-annotated). These findings were illustrated through volcano plot and hierarchical clustering analysis (S2A and S2B Fig). The most highly downregulated circRNAs were 19_58662180_58719032, 7_130721154_130721279, 7_130674581_130681275, 6_41353742_41420524 and 19_58668512_58669244 (circRNA id). Conversely, the most highly upregulated circRNAs included 7_142232409_142233415, 7_130655793_130671489, 7_142232398_142233422, 9_108208070_108212380 and 7_142233169_142233457 (circRNA id). Detailed information regarding all DE circRNAs were presented in S4 Table.

### The sponge and target regulatory elements of DE miRNA in islets

Considering the ability of a single miRNA to target multiple mRNAs and conversely, multiple miRNAs regulating a single mRNA [33, 34], we conducted a target analysis between DE miRNA and DE mRNA pairs. Using the miRanda software, 238 targeted DE mRNAs of the 14 DE miRNAs were predicted, and these mRNAs are expected to be specially regulated by the corresponding miRNAs, as depicted in the network of Fig 3C.

Based on GO enrichment analysis of the targeted DE mRNAs regulated by DE miRNAs, several significant enrichments were observed across categories of molecular functions (MF), cellular components (CC), and biological processes (BP). Remarkably enriched among the MF category were heparan sulfate binding, zymogen binding, alpha-amylase activity, alpha-amylase activity (releasing maltohexaose), and lipoteichoic acid binding category. Within the CC category, collagen-containing extracellular matrix, and extracellular matrix were significantly enriched. The most notably enriched BP category was the induction of bacterial agglutination (Fig 3D).

We then conducted KEGG enrichment analysis to identify the principal biochemical metabolic pathways and signal transduction pathways related to specific mRNA. Among the top 20 pathways, pancreatic secretion, calcium signaling pathway, type 2 diabetes mellitus, and insulin secretion are the significantly enriched pathway associated with in diabetes progression (Fig 3E).

Of particular note, certain DE mRNAs enriched in calcium signaling pathway and type 2 diabetes mellitus pathway play a fundamental in the process of insulin secretion. Specifically, compared to the control group, the expression of *Cacna1c* and *Cacna1b*, which was reported to be downregulated in hypertriglyceridemia subjects with decreased insulin secretion [35], was decreased in HFD mice. Additionally, HFD resulted in the downregulation of *Hk2*, a glycolytic enzyme gene known to promotes insulin secretion [36], and *Cckar*, a crucial receptor stimulating insulin secretion [37]. Furthermore, *Camk2d*, a protein coding gene belonging to the calcium/calmodulin-dependent protein kinase subfamily, which plays a key role in GSIS [38], also exhibited reduced expression in the HFD islets. Taken together, the reduced expression of *Cacna1c*, *Cacna1b*, *Hk2*, *Cckar*, and *Camk2d* may to some extent account for the defective insulin secretion observed in HFD mice. Our subsequent bioinformatics analysis identified a few core regulatory axes, including miR-6948-5p/*Cacna1c*, miR-6964-3p/*Cacna1b*, miR-216a-3p/*Cacna1b*, miR-6948-5p/*Cacna1b*, miR-670-3p/*Cacna1b*, miR-3572-5p/*Hk2*, miR-3572-5p/*Cckar*, miR-670-3p/*Camk2d* and miR-677-5p/*Camk2d* (Fig 3C). Therefore, miR-6948-5p, miR-6964-3p, miR-216a-3p, miR-670-3p, miR-3572-5p, and miR-677-5p exhibit potential regulatory roles in β-cell function within the context of T2DM. The miRNA–mRNA network involved in insulin secretion pathway is depicted schematically in Fig 6A.

Our study also identified several pairs of DE miRNAs and their target mRNAs which linked to the regulation of β-cell mass. Specifically, the FK506-binding protein 51 (encoded by *FKBP5* gene) emerged as a crucial regulator for T2DM, with its inhibition known to protect β-cell survival via *AKT/FOXO1* signaling [39]. Our results revealed that *FKBP5* was downregulated in HFD mice compared with CD group, indicating a pro-survival mechanism mediated by inhibition of *FKBP5* against inflammatory stress in T2DM. In addition, *Foxo3*, a key mediator of apoptosis [40], was found to be downregulated in HFD mice. Moreover, our results indicated a decreased expression of *RIPK1* in the HFD group compared to control mice. Previous evidence suggested that *RIPK1*-deficient β-cells are protected from *TNFα*-induced cell death and caspase activation [41]. Hence, the reduced expression of *FKBP5*, *Foxo3*, and *RIPK1* in our study highlights a potential mechanism against β-cell apoptosis. Previous research have shown that the loss of *Smad2* enhances the expression of proliferative genes in β-cell [42]. Consistently, we also observed a significant decrease in *Smad2* expression level in HFD islets. To sum up, the decreased expression of *FKBP5*, *Foxo3*, *RIPK1* and *Smad2* participated in the pathway regulating β-cell mass in HFD mice. Based on this, our target prediction suggested that miR-216a-3p/*FKBP5*, miR-1188-5p/*FKBP5*, miR-670-3p/*Foxo3*, miR-677-5p/*RIPK1*, miR-802-3p/*Smad2* could represent key regulatory axes, and these miRNAs may serve as potential novel therapeutic targets to promote β-cell mass in T2DM. Fig 6B provided a schematic representation of the miRNA-mRNA network involved in β-cell mass.

### The target regulatory elements of DE lncRNA in islets

Using the DE lncRNA data, we proceeded to predict the target genes for the DE IncRNAs among the differentially expressed mRNAs (mRNAs that were misregulated in the HFD). In our analysis, we observed that 9 out of the 179 DE lncRNAs displayed significant correlation with 10 DE mRNAs in close genomic proximity (cis-correlation), as depicted in Fig 4C. While most of these DE lncRNAs correlated with a single cis-located mRNA, two lncRNAs, namely chr8:94166252–94173140 and ENSMUST00000176781, were found to be potentially associated with two cis target mRNAs each (Fig 4C). These putative target genes were further subjected to GO and KEGG pathway analyses to explore their possible functional connections with the DE lncRNAs.

Based on GO enrichment analysis of the targeted DE mRNAs of DE lncRNAs, the most remarkably enriched term was IgM binding within the MF category. Within the Cellular Component (CC) and Biological Process (BP) categories, the most enriched terms were keratohyalin granule and mammary gland bud morphogenesis, respectively (Fig 4D).

The function of the predicted target DE mRNAs of the identified DE lncRNAs was also assessed through KEGG pathway analysis. The top 20 most enriched pathways, as depicted in Fig 4E, notably included apoptosis—multiple species. As regards to the apoptosis pathway, *caspase9* was identified as the predicted mRNA regulated by ENSMUST00000176781. The reduced expression of caspase9 in HFD islets potentially inhibits apoptosis in β-cell [43, 44], facilitating increased β-cell mass. Therefore, lncRNA ENSMUST00000176781 might represent a novel therapeutic target to promote β-cell mass increase in T2D (Fig 6B).

### Characterization of lncRNA–miRNA–mRNA network reveals potential functional ceRNAs in islets

In addition to mRNAs, several studies have suggested that lncRNAs and circRNAs could also be targeted by miRNAs. These ncRNAs, known as ceRNAs, function by regulating other RNA transcripts through the competition for shared miRNAs [29]. To gain a better understanding of how mRNA expression is regulated by lncRNAs/circRNAs through their interaction with miRNAs, we constructed a ceRNA regulatiory network involving 7 DE miRNAs, 15 DE lncRNAs and 38 DE mRNAs. While numerous DE circRNAs were identified, a ceRNA molecular network involving circRNA–miRNA–mRNA interactions could not be constructed.

**Fig 5:**
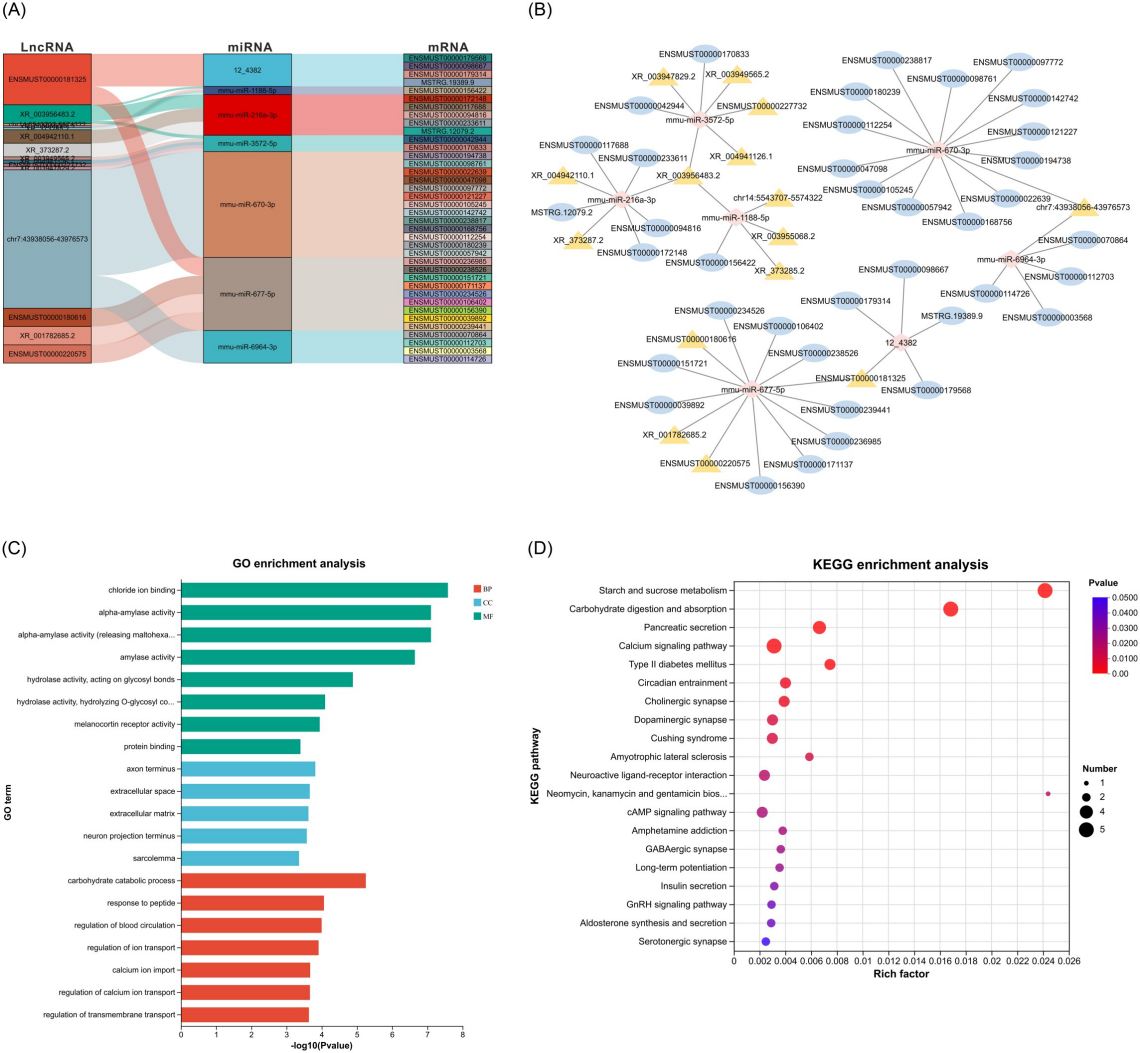
The ceRNA network of lncRNAs–miRNAs–mRNAs and its functional analysis. (A) Sankey diagram for the ceRNA network in islets. Each rectangle represents a gene, and the connection degree of each gene is displayed based on the size of the rectangle. (B) A ceRNA visual network including 38 DE mRNAs, 15 DE lncRNAs, and 7 DE miRNAs. The diamond represents DE miRNAs, ellipses represent DE mRNAs, and triangles represent DE lncRNAs. (C) GO enrichment analysis of the DE mRNAs in ceRNA network. (D) KEGG enrichment analysis of the DE mRNAs in ceRNA network.

The lncRNA–miRNA–mRNA network was constructed and visualized using “ggalluvial” R package and then imported into the Cytoscape software, enabling the assembly of the regulatory ceRNA network (Fig 5A and 5B). This ceRNA network comprises three parts, each centered on 12_4382, miR-1188-5p, miR-216a-3p, miR-3572-5p, miR-670-3p, miR-677-5p, and miR-6964-3p.

**Fig 6:**
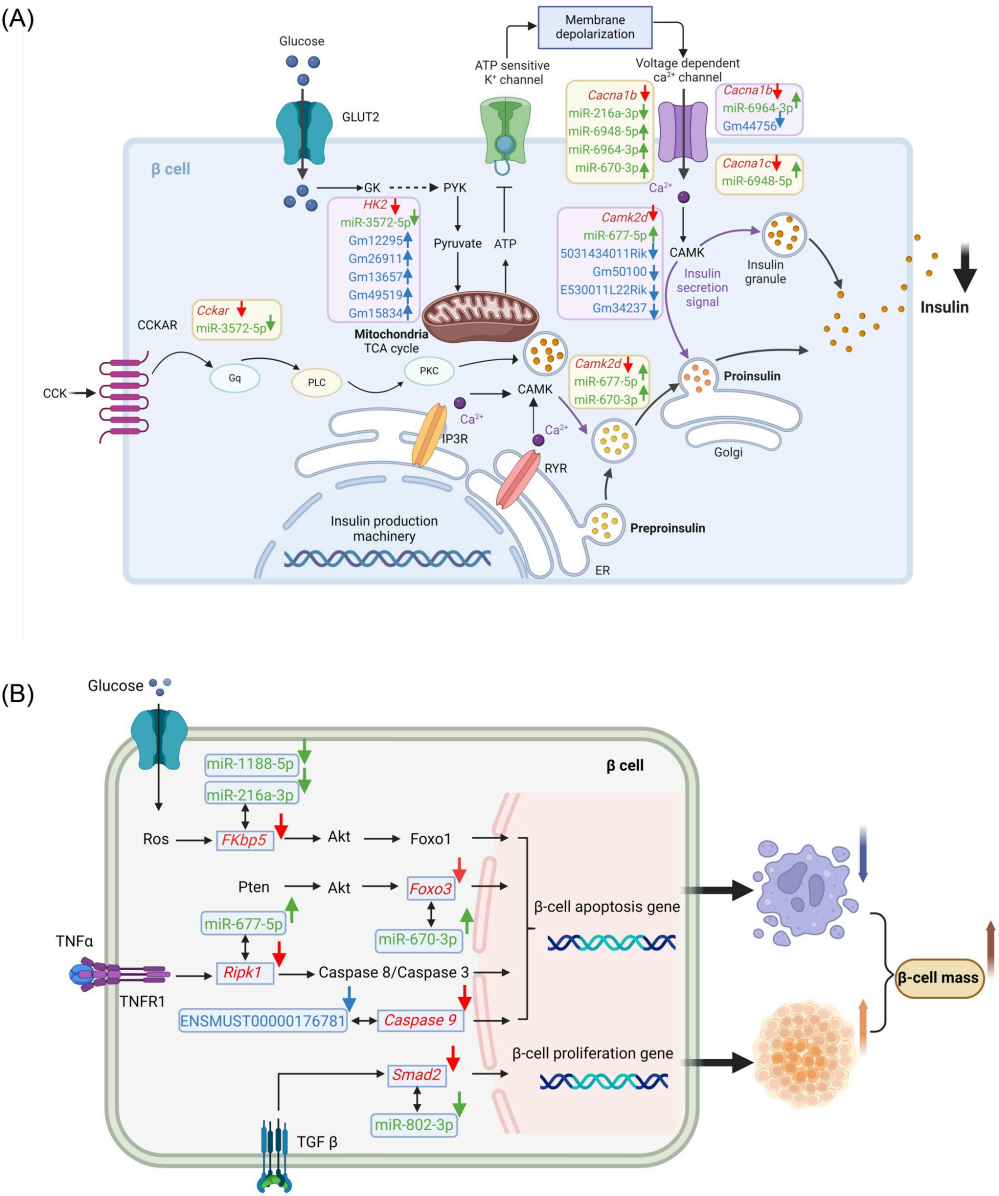
Pathway dynamics of islets during diabetes progression. (A) The schematic representation illustrating the physiology and key ceRNAs involved in impaired insulin secretion of HFD β-cell. mRNAs are colored in red, miRNAs are colored in green, and lncRNAs are colored in blue. The expression change in HFD mice were indicated with arrows. Created with BioRender.com. (B) The diagrammatic representation depicted an overview of LncRNA/miRNA-mRNA change toward increased β-cell mass of HFD mice. mRNAs are colored in red, miRNAs are colored in green, and lncRNA is colored in blue. The expression change in HFD mice were indicated with arrows. Created with BioRender.com.

Thirty-eight mRNAs in the lncRNA–miRNA–mRNA network were subjected to GO and KEGG pathway enrichment analyses. Fig 5C highlighted significantly enriched terms by the mRNAs in the ceRNA network, including chloride ion binding within the MF category, axon terminus in the CC category, and carbohydrate catabolic process in the BP category (Fig 5C). As presented in Fig 5D, KEGG pathway enrichment analysis showcased the top 20 pathways related to these genes. We specifically looked into calcium signaling pathway, T2DM pathway, and insulin secretion pathway, all closely associated with diabetes. In miRNA-mRNA networks, the reduced expression of *Hk2*, regulated by miR-3572-5p, may also be influenced by lncRNAs Gm12295 (gene id:ENSMUSG00000085162, transcript id: XR_003949565.2), Gm26911 (gene id:ENSMUSG00000097834, transcript id: XR_003956483.2), Gm13657 (gene id:ENSMUSG00000086813, transcript id: XR_004941126.1), Gm49519 (gene id: ENSMUSG00000115756, transcript id: ENSMUST00000227732), and Gm15834 (gene id: ENSMUSG00000085054, transcript id: XR_003947829.2). Similarly, the lowered level of *Camk2d* expression, which regulated by miR-677-5p, might be affected by lncRNA 5031434O11Rik (gene id: ENSMUSG00000097885, transcript id: ENSMUST00000180616), Gm50100 (gene id: ENSMUSG00000117696, transcript id: XR_001782685.2), E530011L22Rik (gene id: ENSMUSG00000097820, transcript id: ENSMUST00000181325) and Gm34237 (gene id: ENSMUSG00000113330, transcript id: ENSMUST00000220575). In addition, lncRNA Gm44756 (gene id: ENSMUSG00000108432, transcript id: chr7:43938056–43976573) might exerts an effect on the downregulation of *Cacna1b* by competing for the corresponding miR-6964-3p. The depiction in Fig 6A highlights the changes in the expression of these ceRNAs within the three established lncRNA–miRNA–mRNA networks, offering insight into the impaired insulin secretion observed during the diabetic progression in HFD mice.

## Discussion

The development of T2DM is a multifaceted process influenced by interaction between genetic inheritance, environmental exposures, obesity, and sedentary lifestyles [15, 45, 46]. Long-term overnutrition is a key contributor to the development of T2DM. To simulate the impact of high energy intake on diabetic progression, we developed an obesity-related diabetic mouse model through a 24-week HFD. As compared to CD mice, those on HFD displayed substantial impairments in glucose regulation, compromised islet function, and exhibited signs of liver steatosis. Through whole transcriptomic sequencing of pancreatic islets in both the 24-week HFD and CD mice, DE miRNA, IncRNAs, cirRNA and their targeted DE mRNAs identified in our study reveal association with reduced insulin secretion, increased β-cell mass, and several other biological progresses. Additionally, our research sheds light on the potential role of non-coding RNA-mediated networks in β-cells, encompassing interactions such as miRNA–mRNA, lncRNA–mRNA, and lncRNA–miRNA–mRNA in the development of T2DM.

Insulin is one of the most important anabolic hormone responsible for regulating glucose and energy homeostasis [47]. Since Anderson et al. [48] and Grodsky et al. [49] first provided pivotal evidence for the canonical model of GSIS around 1950s, significant efforts and progress have led to the identification of critical components within the β-cell metabolic signaling machinery for GSIS. Although the crucial physiological role of glucokinase (*GK*) in β-cell glucose sensing has been extensively documented [50], Hexokinase (*HK*), another glycolytic enzyme in β-cell has received relatively less attention. Interestingly, studies have reported that the upregulation of *HK* in β-cells causes a leftward shift of the normal concentration-dependent activation of GSIS, essentially reducing the threshold for glucose sensing [51]. In our study, we observed that several lncRNAs (Gm12295, Gm26911, Gm13657, Gm49519, and Gm15834) were upregulated in HFD islets and computationally predicted to bind miR-3572-5p, which might influence *HK2* expression. The potential downregulation of *HK2* in HFD mice could theoretically elevate the threshold for β-cell glucose sensing, potentially contributing to impaired insulin secretion, particularly under low glucose conditions.

β-cells exhibit electrical excitability, with voltage-gated Ca^2+^ channels (Ca_v_ channels) playing a pivotal role in the process of insulin secretion [52]. Beyond their crucial function in regulating insulin exocytosis, Ca_v_ channels in β-cell significantly contribute to cell development, survival, and growth [52, 53]. Inappropriate regulation of Ca_V_ channels within β-cell can lead to cellular dysfunction and, in severe cases, may result increased mortality rates associated with both type 1 and type 2 diabetes [52, 54]. *Cacna1b* encodes a subunit responsible for high voltage-gated Ca^2+^ channel activity which acts as a source of Ca^2+^ required for excitation-secretion coupling [55]. Meanwhile, *Camk2d* encodes Ca^2+^ calmodulin-dependent protein kinase II, which also plays a significant role in mediating the effect of Ca^2+^ cations on insulin exocytosis [56]. Our data revealed dysregulation of lncRNA–miRNA–mRNA axes, such as Gm44756/miR-6964-3p/*Cacna1b* and 5031434O11Rik-Gm50100-E530011L22Rik-Gm34237/miR-677-5p/*Camk2d*, could be responsible for the defective Ca^2+^ influx and reduced granule release in HFD islets. Taken together, these lncRNAs/miRNAs-associated ceRNA crosstalks provide new insights into the regulation of insulin secretion and offers potential therapeutic targets for addressing impaired glucose homeostasis.

The balance between pancreatic β-cell apoptosis and proliferation is vital for maintaining the β-cell mass [57]. In response to insulin resistance triggered by overnutrition, β-cells are able to compensate by increasing mass through enhanced proliferation and hypertrophy. While previous studies by Mosser *et al*. reported rapid β-cell proliferation occurs within 3 days of HFD feeding [58], our immunofluorescent findings demonstrated a slower rate of proliferation even at 24 weeks of HFD (Fig 1G and 1I). Consistently, our whole transcriptomic sequencing data revealed downregulation of several DE mRNAs which targeted by DE miRNAs or DE lncRNAs in HFD islets, such as *FKBP5*, *Foxo3*, *RIPK1*, *Smad2 and Caspase9*. The reduced expression of these genes could protect β-cell from inflammatory stress and apoptosis, and enhance proliferation, further contributing to the observed increase in β-cell mass.

The novelty of our study is constructing a ncRNA-associated network to investigate the functional and morphological changes in islets of diet-induced obesity and diabetes model. The whole transcriptomic sequencing and comprehensive analyses that we conducted regarding the interactions of miRNA–mRNA, lncRNA–mRNA, and lncRNA–miRNA–mRNA could provide valuable insights into the underlying mechanisms associated with overnutrition, β-cell dysfunction, and compensatory responses in the context of T2DM. Understanding these interactions is crucial in identifying potential therapeutic targets for more effective T2DM management.

The limitations of the study are acknowledged as follows: Our study showed the development of NAFLD and insulin resistance in HFD model, but we haven’t performed a whole transcriptomic sequencing on liver cells in HFD mice to investigate the associated pathways. Given that liver can regulate β-cell function through various metabolic processes and non-coding RNAs [59, 60], conducting liver RNA sequencing simultaneously could offer additional valuable insights into the interplay between the liver and the islets. Furthermore, the predicted targets of DE miRNAs should be further confirmed using methods such as dual luciferase reporter assays, RNA-binding protein immunoprecipitation, and validation at the protein level. In addition, conducting gain- and loss-of-function experiments in future studies is crucial to provide direct experimental evidence of their functional impact. These steps would provide more concrete evidence and strengthen the outcomes of our study.

## Supporting information

S1 Fig HFD induced fatty liver, hyperlipidemia, and hepatic toxicity. (A-B) Representative images of H&E (A) and Oil-red O staining (B) of liver sections. Original magnification ×50 and ×200. (C-G) The average serum TC, TG, LDL, AST and ALT levels of HFD and CD mice. (n≥ 14 mice /group). Data presented as mean ± SEM. **p<0.01, ***p<0.005, **** p<0.001.
(PDF)

S2 Fig The expressional pattern of differentially expressed circRNAs from HFD and CD islets. (A) Differentially expressed circRNAs were exhibited by volcanoplot. (B) Differentially expressed circRNAs were exhibited by clustering analysis.
(PDF)

S1 Table Differentially expressed mRNAs. (CSV)

S2 Table Differentially expressed miRNAs. (CSV)

S3 Table Differentially expressed lncRNAs. (CSV)

S4 Table Differentially expressed circRNAs. (CSV)
