# Supplementary figures and images for: Whole transcriptome sequencing analyses of islets reveal ncRNA regulatory networks underlying impaired insulin secretion and increased β-cell mass in high fat diet-induced diabetes mellitus
Source: PLoS One. 2024 Apr 1;19(4):e0300965. doi: 10.1371/journal.pone.0300965 (PMC10984535; doi:10.1371/journal.pone.0300965)

(A)

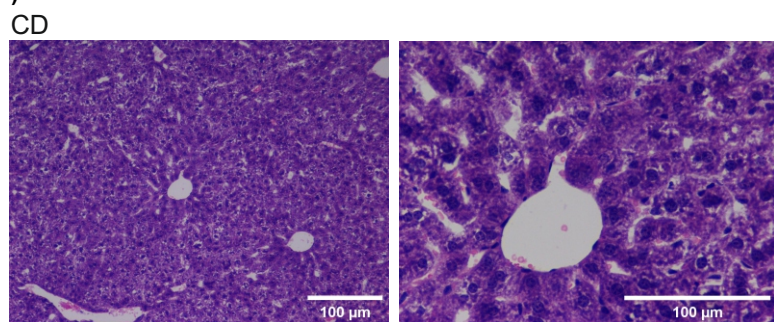

(B)

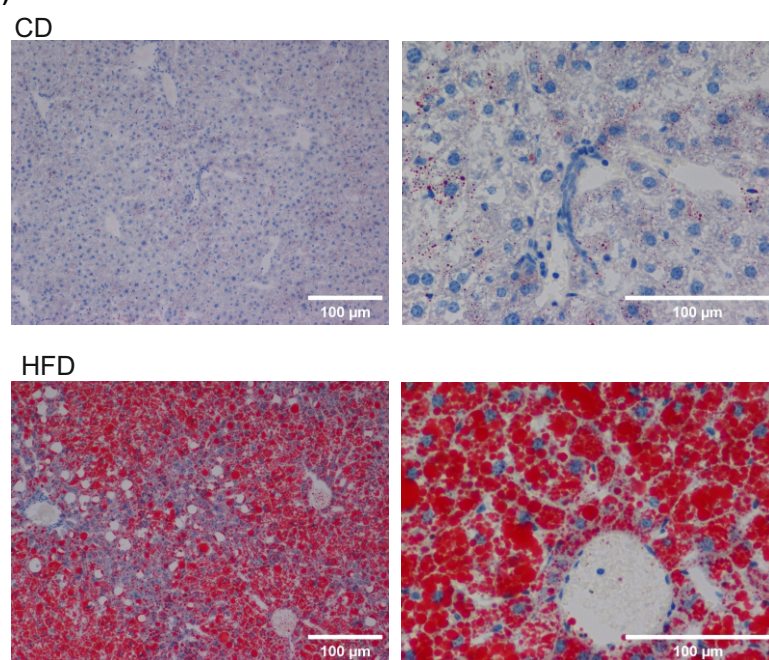

(C)

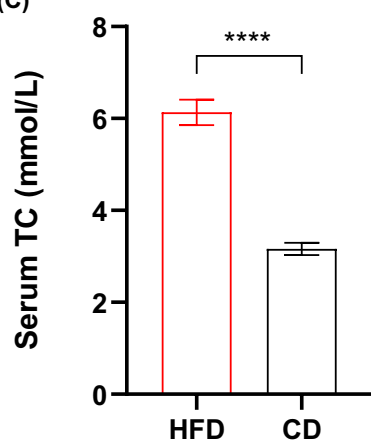

(D)

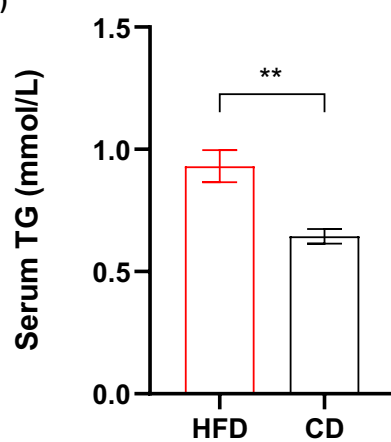

(E)

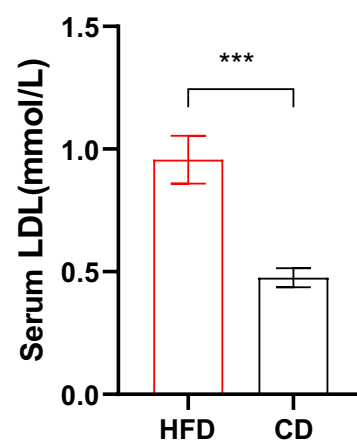

(F)

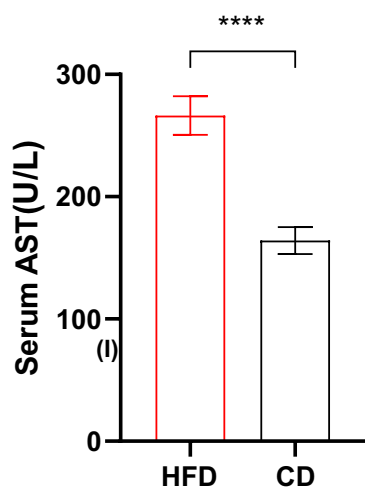

(G)

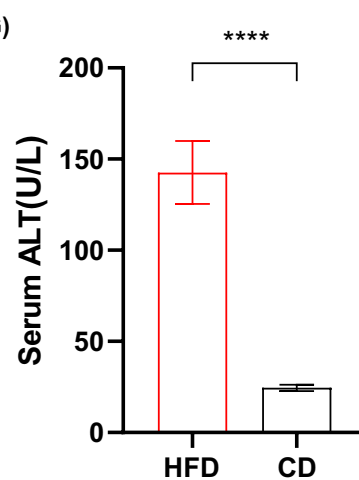

Supplement: S1 Fig — (A-B) Representative images of H&E (A) and Oil-red O staining (B) of liver sections. Original magnification ×50 and ×200. (C-G) The average serum TC, TG, LDL, AST and ALT levels of HFD and CD mice. (n≥ 14 mice /group). Data presented as mean ± SEM. **p<0.01, ***p<0.005, **** p<0.001. (PDF) [file pone.0300965.s001.pdf]

(A)

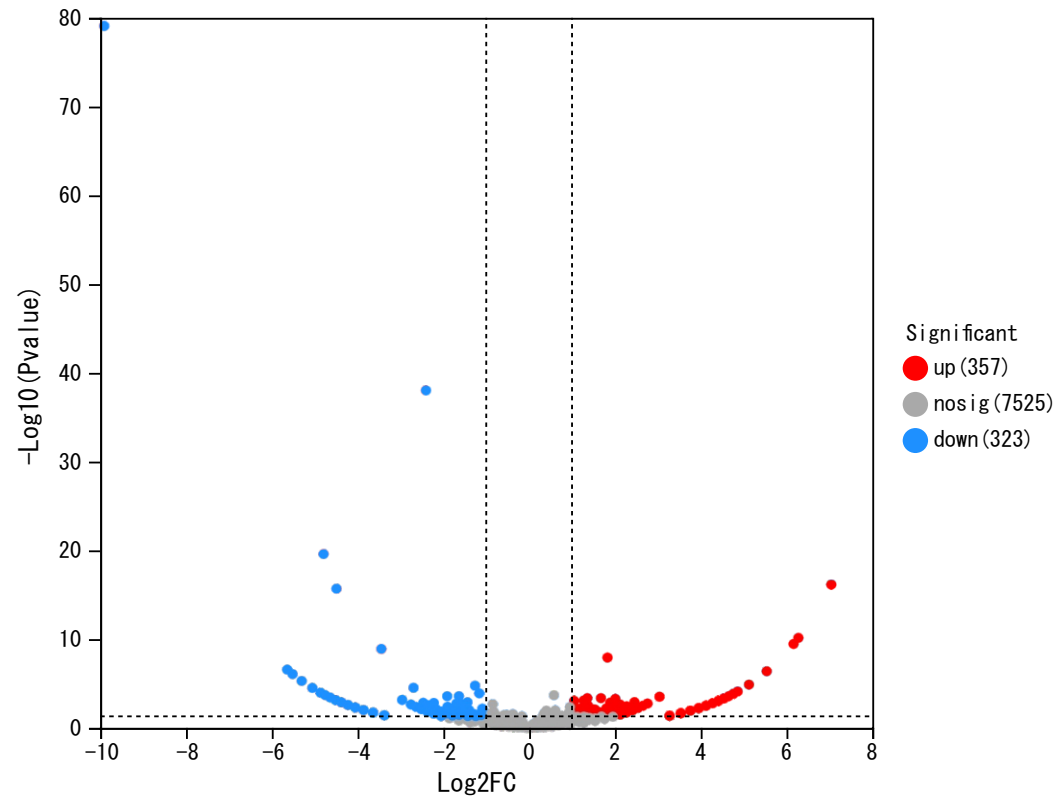

(B)

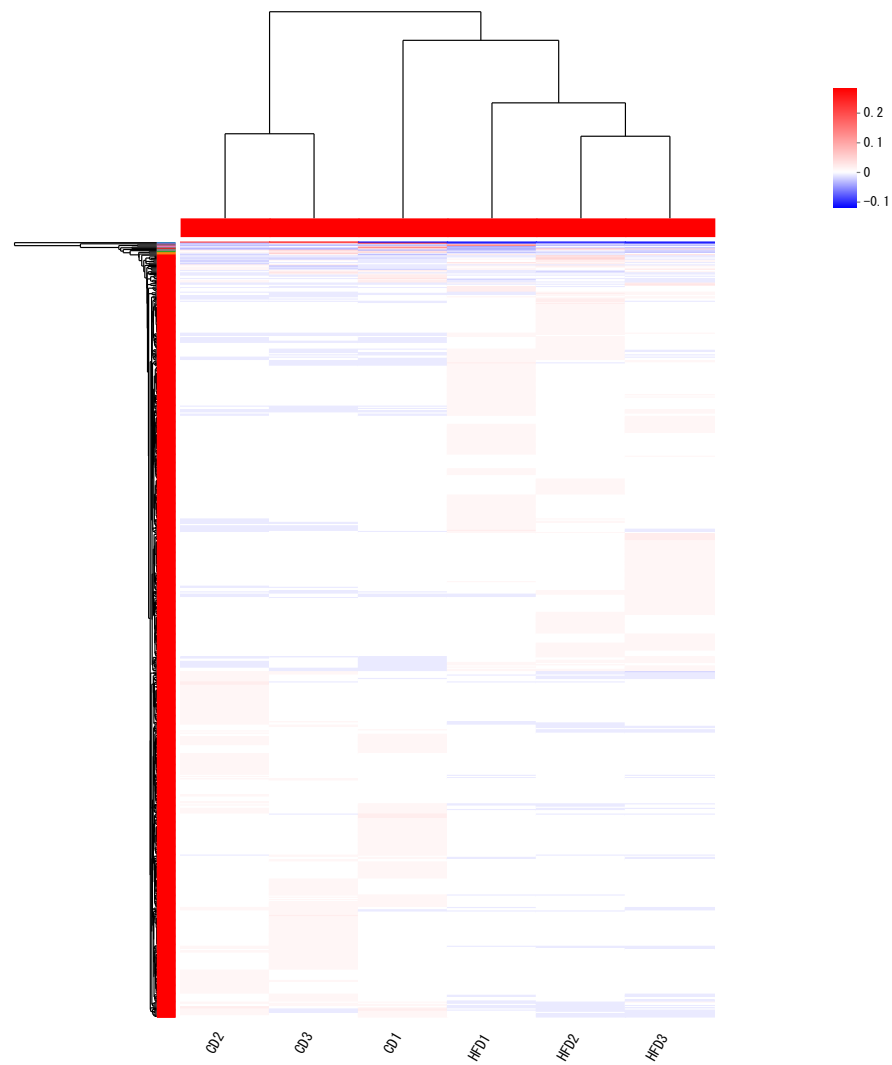

Supplement: S2 Fig — (A) Differentially expressed circRNAs were exhibited by volcanoplot. (B) Differentially expressed circRNAs were exhibited by clustering analysis. (PDF) [file pone.0300965.s002.pdf]
